# Identification of biomarkers for the accurate and sensitive diagnosis of three bacterial pneumonia pathogens using in silico approaches

**DOI:** 10.1186/s12860-020-00328-4

**Published:** 2020-11-20

**Authors:** Olalekan Olanrewaju Bakare, Marshall Keyster, Ashley Pretorius

**Affiliations:** 1 Bioinformatics Research Group, Biotechnology Department, University of the Western Cape, Cape Town, 7535 South Africa; 2 Environmental Biotechnology Laboratory, Biotechnology Department, University of the Western Cape, Cape Town, 7535 South Africa

**Keywords:** Antimicrobial peptides, Bacteria, Databases, Algorithms, Pathogens, Diagnostics, Receptors, Protein and ligands

## Abstract

**Background:**

Pneumonia ranks as one of the main infectious sources of mortality among kids under 5 years of age, killing 2500 a day; late research has additionally demonstrated that mortality is higher in the elderly. A few biomarkers, which up to this point have been distinguished for its determination lack specificity, as these biomarkers fail to build up a differentiation between pneumonia and other related diseases, for example, pulmonary tuberculosis and Human Immunodeficiency Infection (HIV). There is an inclusive global consensus of an improved comprehension of the utilization of new biomarkers, which are delivered in light of pneumonia infection for precision identification to defeat these previously mentioned constraints. Antimicrobial peptides (AMPs) have been demonstrated to be promising remedial specialists against numerous illnesses. This research work sought to identify AMPs as biomarkers for three bacterial pneumonia pathogens such as *Streptococcus pneumoniae, Klebsiella pneumoniae, Acinetobacter baumannii* using in silico technology. Hidden Markov Models (HMMER) was used to identify putative anti-bacterial pneumonia AMPs against the identified receptor proteins of *Streptococcus pneumoniae, Klebsiella pneumoniae,* and *Acinetobacter baumannii*. The physicochemical parameters of these putative AMPs were computed and their 3-D structures were predicted using I-TASSER. These AMPs were subsequently subjected to docking interaction analysis against the identified bacterial pneumonia pathogen proteins using PATCHDOCK.

**Results:**

The in silico results showed 18 antibacterial AMPs which were ranked based on their E values with significant physicochemical parameters in conformity with known experimentally validated AMPs. The AMPs also bound the pneumonia receptors of their respective pathogens sensitively at the extracellular regions.

**Conclusions:**

The propensity of these AMPs to bind pneumonia pathogens proteins justifies that they would be potential applicant biomarkers for the recognizable detection of these bacterial pathogens in a point-of-care POC pneumonia diagnostics. The high sensitivity, accuracy, and specificity of the AMPs likewise justify the utilization of HMMER in the design and discovery of AMPs for disease diagnostics and therapeutics.

## Background

Pneumonia disease is an infection of the lung parenchyma and it is one of the major causes of critical illness throughout the world [1]. Approximately, 150 million cases occur every year among children under 5 years of age and the elderly, resulting in approximately 20 million hospitalizations. Treatment of the disease is hampered by a lack of sensitive diagnosis [2] as biomarkers which hitherto have been identified for its diagnosis, lack specificity because they are implicated in other diseases such as pulmonary tuberculosis and Human Immunodeficiency Virus (HIV). The growing problems of diagnosis of this disease and the difficulty of resistance to conventional antibiotics, there is growing attention in the laboratory and pharmacological application of antimicrobial peptides to detect and treat infections.

Five diagnostic biomarkers have been linked to the diagnosis of pneumonia, namely: C-reactive protein (CRP), Procalcitonin (PCT), a Soluble triggering receptor expressed on myeloid cells-1 (STREM-1), CD163, and High Mobility Group Box-1(HMGB-1). CRP and PCT have been proven useful in diagnosis as they are produced in considerably high concentration but there is ambiguity in their specificity towards pneumonia because they can be produced in response to other inflammatory stimuli in the neuron, atherosclerotic plaques, myocytes, and lymphocytes [3]; whilst, the mechanism regulating their syntheses at these sites is not clearly understood [4]. There are other biomarkers that are currently being studied for their probable link with pneumonia diagnosis; these include copeptin, cortisol, endotoxin, pro-adrenomedullin, amongst others, yet their roles in pneumonia are not understood [3].

Apart from this, the methods of detection of the biomarkers implicated in pneumonia disease have been constantly advanced, ranging from poor sensitivity of blood cultures [5], the inability of X-ray to identify the causative pathogen [6], overwhelming lack of sensitivity and specificity of the polymerase chain reaction [7], inability to identify only a few bacterial pneumonia pathogens in matrix-assisted laser desorption or ionization-time of flight [8], to expensive and lack of sensitivity of immunofiltration and turbidimetric immunoassay [9]. The implication of the use of these methods for pneumonia biomarkers identification is the generation of false-negative and false-positive results in patients [6]. It is therefore important to explore other more reliable methods with improved sensitivity and accuracy towards pneumonia.

Furthermore, antimicrobial peptides (AMPs) are part of the innate immune systems that shield multicellular organisms from a diverse spectrum of micro-organisms [10]. They have sequence composition that is family-specific which can be used to discover and design novel ones [11]. They generally have therapeutic efficacy to microbial targets when compared to conventional antibiotics with other compensatory advantages ranging from expansive broad-spectrum activities, low toxicity, and low resistance by microbes. These significant advantages give them huge popularity and attention as novel antimicrobial agents. Previous research has also reported the use of AMPs as diagnostic agents against p24 of HIV [12] which were subsequently validated for point-of-care (POC) use [1]. In this research, a similar effort would be made to use AMPs as biomarkers against pneumonia receptors.

Apart from this, several in silico tools exist to identify novel AMPs that are fast, cheap, and less-labor intensive thus speeding up the discovery process. Among them is the Hidden Markov Models (HMMER) software which has several modules to perform optimally using several command lines. The high sensitivity of the HMMER profiles is due to the combination of the scoring system in the form of E-value. The E-value gives more information about the probability of that predicted AMPs to be true positive or false negative AMPs [13, 14]. The appropriate use of the HMMER algorithm enables a more sophisticated search of novel peptides through scanning of the proteome.

This research work aimed to generate parameters for AMPs that could be used as novel biomarkers for bacterial pneumonia diagnosis with a view to speeding up accurate diagnosis using *in-silico* technology such as HMMER. This is because the discovery of more biomarkers is imperative, using bioinformatics for example, for more accurate diagnosis and assurance of functional specificity to ameliorate the aforementioned problems associated with pneumonia biomarkers. With this, there is a potential promising perspective to reduce the problems of indiscriminate overuse, toxicity due to the wrong prescription, bacterial resistance, scarcity, and the high cost of existing antibiotics.

## Results

### Retrieval of anti-bacterial AMPs (BAP-AMPs)

In this section, experimentally validated AMPs were retrieved from various databases where literature mining revealed that CAMP, APD, and BACTIBASE had 155, 9, and 4 experimentally validated bacterial anti-pneumonia antimicrobial peptides (BAP-AMPs) respectively. BAP-AMPs against the pathogens *Klebsiella pneumonia* totaled 140 peptides*, Streptococcus pneumoniae* totalled 16*,* and *Acinetobacter baumannii* totaled 12 peptides combined from the various databases (see Table 1). These experimentally validated anti-pneumonia AMPs were derived from bacteria, arthropods, Mammalia Amphibia, nematode, Pisces, Arachnida, aves, plants, reptilian, fungi, and viruses with the numbers for each division indicated in Table 1. The peptide total in Table 1 represents unique peptides for each organism following the removal of duplicates.

**Table 1:** Profile creation by HMMER

Profiles	Training Datasets	Testing Datasets	Total
**AB**	9	3	12
**SP**	12	4	16
**KP**	105	35	140

Legend: *AB* Anti-Acinetobacter baumannii*, SP* Anti-Streptococcus pneumoniae, *KP*: Anti-Klebsiella pneumoniae

### Profile creation using HMMER

The first step in the profile creation pipeline was the random grouping of the different classes into ¾ and ¼ of the experimentally validated AMPs. The ¾ is the training dataset, needed to train the algorithm to test whether the functionally significant amino acid consensus is conserved. After this, multiple alignments were generated using HMMER ClustalW.

### Independent testing of the created profiles

Each created profile was tested against a positive dataset which represented about a quarter of the dataset, from which the training dataset used for the construction of the respective profiles was derived as well i.e. the profiles created using training dataset must have the ability to recognize and identify this subset of AMPs. Since experimentally verified AMPs were used, the assumption is that the profiles constructed should be able to identify other sequences with the exact same activity and discriminate those that have no anti-pneumonia activity from same pathogen. The trained profiles were also scanned against a negative control dataset, from UNIPROT database (http://www.uniprot.org) made up of random fragments of 17,236 neuropeptides, which had no recorded anti-pneumonia activity.

### Evaluation of the independent testing results

The independent testing of the profiles were evaluated using the true positive (TP), false positive (FP), true negative (TN), and false-negative (FN). A cut-off E-value of 0.05 was applied to the HMMER algorithm to strengthen the ability of the profile to discriminate between the true positive anti-pneumonia AMP and false-negative anti-pneumonia AMPs. TP (True positive) represents correctly predicted positive sequences (anti-pneumonia AMPs), TN (True negative) denotes correctly predicted negative sequences (non-anti-pneumonia AMPs), FP (False positive) is the number of non-anti-pneumonia AMPs wrongly predicted as anti-pneumonia AMPs (AP-AMPs), FN (False negative) is the number of anti-pneumonia AMPs wrongly predicted as non-anti-pneumonia AMPs. It was possible to calculate the number of TP AMPs from the total number of input sequences, thus the FP number could be extrapolated with the results shown in Table 2, reflecting the capacity of each profile to distinguish true anti-pneumonia AMPs from false anti-pneumonia AMPs.

**Table 2:** Independent testing of profiles against test and negative datasets

Profiles	TP	FN	TN	FP
**AB**	3	0	17,236	0
**SP**	1	3	17,236	0
**KP**	10	25	17,236	0

Legend: *AB* Anti-*Acinetotobacter baumannii*, *SP* Anti-*Streptococcus pneumoniae*, *KP*, Anti-*Klebsiella pneumoniae*

### Performance measurement of the target specific profiles

After evaluating the ability of the optimized profiles, the performance was calculated with the aim of determining the robustness of each profile, using specificity, sensitivity, accuracy and Matthew’s Correlation Coefficient (MCC), introduced by biochemist Brian W. Matthews in 1975 [15]. The sensitivity, specificity, accuracy and MCC were calculated as reported in Table 3.

**Table 3:** Summary of Performance measurement of the profiles

Models	Sensitivity (%)	Specificity (%)	Accuracy (%)	MCC
AB	100	100	100	1
SP	25	100	99.6	0.50
KP	46	100	99.9	0.63

Legend: *AB* Anti-*Acinetobacter baumannii*, *SP* Anti-*Streptococcus pneumoniae*, *KP* Anti-*Klebsiella pneumoniae*

From the result shown in Table 3, sensitivity values were high in AB of anti-bacterial profiles tested. KP had 46%. All profiles were specific and accurate with significant MCC values. The MCC is considered to give the best performance measurement of models because it incorporates sensitivity, specificity, and accuracy [16]. The specificity results for all profiles were 100%.

High sensitivity values of AB profiles showed correct predictions. The moderate sensitivity of KP could be attributed to the significant overlap in the conserved domain of the AMPs used for their profile construction [17]. However, the relatively low sensitivity of SP could be due to low available AMPs against *Streptococcus pneumoniae* from the databases coupled with a serious overlap in the conserved consensus [18]. The profiles showed very significant accuracy results. Accuracy is a commonly used predictive profile parameter to reduce errors by establishing several misclassified AMPs from both positive and negative datasets. MCC values for all the profiles showed very significant results with the lowest value recorded for SP (0.50). The MCC value ‘0.5 to 1’ corresponds to the perfect prediction, whereas ‘0’ points to a completely random prediction. Thus all profiles indicated correct prediction (AB > KP > SP).

### Proteome sequence databases query and discovery of putative anti-pneumonia AMPs

The discovery stage was to search for novel bacterial and viral anti-pneumonia AMPs against *Acinetobacter baumannii, Streptococcus pneumoniae, Klebsiella pneumoniae* toidentify peptides that had the same signatures/motifs and properties as the input sequences used to build the profiles AB, SP, KP. The matches of the respective profiles to the proteome sequences were also shown with E-values (Table 4). The E-value of 0.05 cut-off was applied to search for putative AMPs.

**Table 4:** Final list of the Anti-bacterial pneumonia AMPs with their sources

S/N	TARGET	ORGANISMS	E-VALUE	SEQUENCE
1	BOPAM-AB1	*Amphibian predicted*	0.0076	FLPI----LKSGLSGLL
2	BOPAM-AB 2	*Amphibian predicted*	0.0091	FFP----LLKSGLSGLL
3	BOPAM-AB3	*Amphibian predicted*	0.014	FFP----LLKFGLFGLL
4	BOPAM-AB4	*Amphibian predicted*	0.02	FFP----LLKFGLSGLL
5	BOPAM-KP 2	*Atta cephalotes*	0.008	MWK----GGLNNGVQCKIDNC
6	BOPAM-KP 3	*Atta cephalotes*	0.009	MWK----LGNNFTVQCKIDNC
7	BOPAM-KP 4	*Atta cephalotes*	0.010	IHH----GGYIPYLKWHLRKK
8	BOPAM-KP 5	*Drosophila erecta*	0.011	ICK----ISGHCSASLKCWFKKR
9	BOPAM-KP 6	*Drosophila mojavensis*	0.013	LAK----AGAEKKKCKELAKK
10	BOPAM-KP 7	*Drosophila erecta*	0.015	LCR----VSGHCSASLKCWRAMK
11	BOPAM-SP1	*Homo sapiens*	6.9e-16	QGRDD----SIMRRRGLTSPCKDINTFIHGNKRSIKAICENKNG
12	BOPAM-SP2	*Felis catus*	5.1e-11	KGRND----SMMERRGLTTPCKDTNTFIHGNKGSIKAICGNKNG
13	BOPAM-SP3	*Bos Taurus*	5.2e-10	FRH----GEYEVHHQKLVFFAEDVSGSNKGFCAIIGLMVGGVVI
14	BOPAM-SP3	*Homo sapiens*	5.3e-10	FRH----GEYEVHHQKLVFFAEDVSGSNKGFCAIIGLMVGGVVI
15	BOPAM-SP4	*Latimeria chalumnae*	1.5e-09	FRH----GEYEVHHQKLVFFAEDVSGSNKGFCAIIGLMVGGIVI
16	BOPAM-SP5	*Felis catus*	6e-09	FRH----GEYEVHHQKLVFFAEDVSGSNKGFCAIIGLMVGGVVI
17	BOPAM-SP 7	*Xenopus amphibians query*	2.9e-07	YRH----GEYEVHHQKLVFFAEEVSGSNKGFCAIIGLMVGGVVI

LEGEND: *S/N* Serial number, *BOPAM-AB1–4* Putative Anti-*Acinetobacter baumannii* AMP, *BOPAM-SP1–8* Putative Anti-*Streptococcus pneumoniae* AMP, *BOPAM-KP2–7* Putative Anti-*Klebsiella pneumoniae* AMP

Scanning the profiles to identify novel anti-bacterial AMPs, profile AB identified 7 AMP sequences that adhered to the E value set which yielded 5 after removing the duplicate. Profile SP identified 58 AMP sequences that adhered to E value set which yielded 8 after removing duplicate while profile KP identified 7 without duplicate. These anti-bacterial peptide sequences were all single domains. These putative AMPs were named by adding BOPAM to the trained profiles from which they were derived (BOPAM-AB, BOPAM-SP, and BOPAM-KP). BLAST analysis was used to eliminate BOPAM-AB5 due to its 100% similarity to moricin whilst BOPAM-KP1 was eliminated due to its 100% similarity to drosomycin.

### Physicochemica**l** properties of the AMPs

The physicochemical properties of the putative AMPs were determined using APD and BACTIBASE to ensure that the identified sequences conform to other known AMPs based on the characteristics measured (Table 5). Physicochemical features such as molecular weight amino acid composition, hydrophobicity, Boman index, net charge, isoelectric potential, and half-life were used to evaluate the anti-bacterial and anti-viral AMPs.

**Table 5:** Physicochemical parameter of the anti-bacterial pneumonia putative AMPs

S/N	Profile	Mass number (Da)	% Hydrophobic	Common amino acid	Net charge	PI	Boman Index (kcal/mol)	Half-life (Hours)
1	BOPAM-AB1	1755.35	52	L	+ 2	10.81	− 1.55	1.1
2	BOPAM-AB2	1789.36	52	L	+ 2	10.81	−1.43	1.1
3	BOPAM-AB3	1909.56	64	L	+ 2	10.81	−2.18	1.1
4	BOPAM-AB4	1849.46	58	L	+ 2	10.81	−1.81	1.1
5	BOPAM-KP2	2370.21	42	NKGV	+ 2	8.70	0.9	30
6	BOPAM-KP3	2478.35	47	NKV	+ 3	8.82	1.18	30
7	BOPAM-KP4	2532.14	28	K	+ 7	10.58	1.5	20
8	BOPAM-KP5	2667.29	39	K	+ 5	9.66	2.3	20
9	BOPAM-KP6	2300.00	38	K	+ 8	10.98	2.28	5.5
10	BOPAM-KP7	2589.16	43	CS	+ 4	8.53	2.0	5.5
11	BOPAM-SP1	5083.36	27	R	+ 6	10.06	3.62	0.8
12	BOPAM-SP2	4903.88	25	G	+ 6	10.00	3.06	1.3
13	BOPAM-SP3	4765.02	45	G	+ 1	6.22	0.47	1.1
14	BOPAM-SP4	4779.05	45	G	+ 1	6.22	0.45	1.1
15	BOPAM-SP5	4779.05	45	G	+ 1	6.22	0.43	1.1
16	BOPAM-SP6	4765.02	45	G	+ 1	6.22	0.47	1.1
17	BOPAM-SP7	4793.07	47	GV	+ 1	6.22	0.36	2.8

LEGEND: *S/N* Serial number, *BOPAM-AB1–5* Putative Anti-*Acinetobacter baumannii* AMP, *BOPAM-SP1–8* Putative Anti-*Streptococcus pneumoniae* AMP, *BOPAM-KP1–2* Putative Anti-*Klebsiella pneumoniae* AMP

The amino acid composition of the AMPs contributes to the molecular weight, since the AMPs are made up of amino acids and can be a distinguishing feature to discriminate between two classes of protein/peptides. The antibacterial pneumonia AMPs have some amino acids which are common to them for discrimination against one another (Table 5). BOPAM-AB1–4 had common amino acid leucine while BOPAM-AB5 had asparagine, glycine, lysine, and valine. BOPAM-KP2 had asparagine; BOPAM-KP3 had lysine and glycine; BOPAM-KP4–6 had lysine while BOPAM-KP7 had cysteine and serine. BOPAM-SP1 had arginine; BOPAM-SP2–6 had glycine while BOPAM-SP7 had glycine and valine. The anti-pneumonia AMPs such as BOPAM-KP4, BOPAM-SP1, and 2, had hydrophobicity less than 30% due to the presence of more polar amino acid residues (Table 5) [19]. All anti-bacterial pneumonia AMPs were positively charged. Anti-bacterial pneumonia AMPs have pI between 6.22 and 12.91 (Table 5). This range of values shows solubility properties for the AMPs despite the variability of charges in acid and alkaline media [20]. The results of the Boman index showed negative values for BOPAM-AB1–4 (Table 5). A negative Boman index has been said to be positively correlated with a more hydrophobic peptide, indicating a high protein binding potential. The half-life results of the anti-bacterial pneumonia AMPs showed that BOPAM-AB1–4 had 1.1 h; BOPAM-KP2–4 had 30 h, BOPAM-KP5 had 20 h while BOPAM-KP6 and 7 had 5.5 h. BOPAM-SP1 had a half-life of 0.8, BOPAM-SP2, and 7 had a half-life of 1.3, BOPAM-SP3–6 had a half-life of 1.1 while BOPAM-SP7 had a half-life of 2.8.

### Retrieval of protein receptors of pneumonia pathogens

This section was carried out to determine the immunogenic proteins of bacterial pneumonia of diagnostic relevance to serve as targets for the novel antimicrobial peptides for the diagnosis of the different pathogens. Several pneumonia proteins such as cell surface receptors and nucleoproteins were identified for the pneumonia pathogens *Acinetobacter baumanni*, *Streptocococcus pneumoniae*, *Klebsiella pneumoniae*. These protein receptors of pneumonia pathogens were retrieved from the protein databank (PDB) in National Centre for Bioinformatics Institute (NCBI) database and are projected to be potentially relevant in the diagnosis of bacterial pneumonia pathogens.

*Acinetobacter baumannii* is an emerging nosocomial pathogen that is resistant to many types of antibiotics, and hence, a fast, sensitive, specific, and economical test for its rapid diagnosis is needed. Analysis of the pneumonia pathogen proteins from APD and BACTIBASE showed that *Acinetobacter baumannii* has an iron-regulated outer membrane receptor protein with molecular weight 85,519.34 Da, isoelectric point 7.55, hydrophobicity 31.22%, charge + 1, instability index 25.61, and Half-life of 30 h in mammals. *Streptococcus pneumoniae* is presumed to be the primary bacterial cause of community-acquired lower respiratory infections and meningitis among children and the elderly in many countries. The laboratory diagnosis of invasive pneumococcal disease continues to rely on culture-based methods from appropriate clinical samples such as blood, pleural fluid, or purulent sputum that have been used for many decades. Pneumolysin was identified for *Streptococcus pneumoniae*, which has a molecular weight of 52,896.42 Da, an isoelectric point of 5.18, hydrophobicity of 33.97%, charge − 14, instability index of 20.69, and Half-life of 30 h in mammals (Table 6). Evidence indicates that *Klebsiella pneumoniae* infections are characterized by a lack of early inflammatory response, thus making detection difficult. However, it is unknown whether *Klebsiella pneumoniae* employs additional factors to modulate host inflammatory responses to escape detection. Results indicated that *Klebsiella pneumoniae* has an iron-regulated outer membrane protein with molecular weight 80,401.89 Da, isoelectric point 4.89, hydrophobicity 32.37%, charge − 24, instability index of 35.81, and Half-life of 30 h in mammals (Table 6).

**Table 6:** Physicochemical Properties of the Retrieved Pneumonia Receptor Proteins

S/N	Anti-Pneumonia proteins	Molecular weight (Da)	Hydrophobicity (%)	Net charge	Instability index	Half-life (hours)
1	AB Iron regulated OMP	85,519.34	31.22	+ 1	25.61	30
7	SP Pneumolysin	52,896.42	33.97	−14	20.69	30
8	KP Iron regulated OMP	80,401.89	32.37	−24	35.81	30

*AB Acinetobacter baumanni*, *SP Streptocococcus pneumoniae KP Klebsiella pneumoniae*

### Final list of the pneumonia receptors

#### Structure prediction of the putative anti-pneumonia AMPs and pneumonia protein receptors

**Fig. 1:**
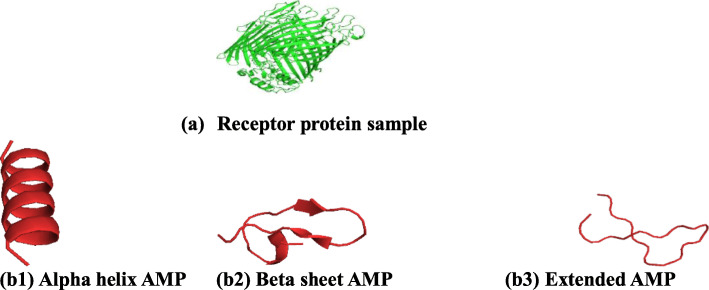
3D structures of the AMPs and pneumonia protein as determined by I-TASSER. 3D structure of (a) *Acinetobacter baumannii* outer membrane protein (b1) alpha helical AMP, (b2) beta sheet AMP, (b3) extended sheet AMP

Representative output images from the I-TASSER server after predicting the 3-D structures of the anti-pneumonia AMPs (ligands) and the protein receptors are indicated in Fig. 1. The results indicate that all AMPs predicted exhibited various secondary structures including α-helices, parallel β-sheet, anti-parallel β-sheet, extended, and loop conformational structures (Fig. 1). The results observed are in line with the various structural conformations exhibited by known AMPs. Examples of known AMPs and their structures include tachyplesin from horseshoe crabs and bovine lactoferricin which have beta-sheet conformations [21]; magainin analog and melittin having alpha-helical conformations [22].

For structure prediction evaluation using I-TASSER (Table 7) several parameters such as C-score, TM-score, and RMSD were used for the prediction of the AMPs and pneumonia protein receptor 3-D structures.

**Table 7:** Quality assessment scores of the predicted 3-D structures of the bacterial pneumonia receptors

S/N	Anti-Pneumonia proteins	C-score	Exp. TM Score	Exp. RSMD
1	AB Iron regulated OMP	−0.18	0.69 ± 0.12	2.8 ± 1.6 Å
2	SP Pneumolysin	1.77	0.96 ± 0.05	3.5 ± 2.4 Å
3	KP Iron regulated OMP	−0.47	0.70 ± 0.12	2.5 ± 1.5 Å

*S/N* Serial number, *AB Acinetobacter baumanni*, *SP Streptocococcus pneumoniae*, *KP Klebsiella pneumoniae*

##### C-Score

C-score is a confidence score for estimating the quality of predicted models by I-TASSER. Its calculation is based on the significance of threading template alignments and the convergence parameters of the structure assembly simulations, which is typically in the range of − 5 to 2, where a C-score within this range of values, signifies a model with high confidence (Zhang, [23]). The results indicate that the C-score of all the predicted 3-D structures for the anti-bacterial pneumonia AMPs and the pneumonia receptor proteins were between the values of − 5 to 2 (see Tables 7, 8, 9, and 10) especially the C-score of the pneumonia receptor proteins. The calculated C-scores of BOPAM-KP2, 3 (Table 9), 4, BOPAM-SP3, 4, and 6 (Table 10) were lower than that of the other AMPs; and could indicate that these molecules did not have an available template for their prediction but were still not randomly predicted (Roy et al., [24]). The lack of templates for the prediction of these molecules can indicate their novelty. A TM-score > 0.5 indicates a model of correct topology and a TM-score < 0.17 means a random similarity. These cut-offs do not depend on the protein length. From the results (Tables 7, 8, 9, and 10), the TM-score of the predicted structures of the AMPs were higher than the cut-off value of 0.5, except for BOPAM-KP2 with a TM-score of 0.49 ± 0.15. BOPAM-KP2 also had a lower than expected C-score adding to the notion that there are no templates for this particular molecule at the time of the study. Although there is not a defined RMSD value for 3-D structure prediction, an RMSD value of 2–4 Å is considered good and an RMSD ≤1 Å is considered ideal.

**Table 8:** Quality assessment scores of the predicted 3-D structures of the putative anti-*Acinetobacter baumannii* AMPs

S/N	Putative AMP	C-Score	Exp. TM Score	Exp. RSMD
1	BOPAM-AB1	−0.51	0.65 ± 0.13	1.6 ± 1.4 Å
2	BOPAM-AB2	−0.49	0.65 ± 0.13	1.6 ± 1.4 Å
3	BOPAM-AB3	−0.27	0.68 ± 0.12	1.2 ± 1.2 Å
4	BOPAM-AB4	−0.32	0.67 ± 0.13	1.3 ± 1.3 Å

**Table 9:** Quality assessment scores of the predicted 3-D structures of the putative anti-*Klebsiella pneumonia* AMPs

S/N	Putative AMP	C-Score	Exp. TM Score	Exp. RSMD
1	BOPAM-KP2	−1.84	0.49 ± 0.15	4.5 ± 3.0 Å
2	BOPAM-KP3	−1.55	0.52 ± 0.15	3.9 ± 2.7 Å
3	BOPAM-KP4	−1.52	0.53 ± 0.15	3.9 ± 2.6 Å
4	BOPAM-KP5	−0.18	0.69 ± 0.12	1.6 ± 1.4 Å
5	BOPAM-KP6	−0.06	0.71 ± 0.12	1.2 ± 1.2 Å
6	BOPAM-KP7	−0.39	0.66 ± 0.13	1.9 ± 1.6 Å

**Table 10:** Quality assessment scores of the predicted 3-D structures of the putative anti-*Streptococcus pneumonia* AMPs

S/N	Putative AMP	C-Score	Exp. TM Score	Exp. RSMD
1	BOPAM-SP1	−0.05	0.71 ± 0.12	2.5 ± 1.9 Å
2	BOPAM-SP2	−0.00	0.71 ± 0.11	2.4 ± 1.8 Å
3	BOPAM-SP3	−1.74	0.50 ± 0.15	5.8 ± 3.6 Å
4	BOPAM-SP4	−1.70	0.51 ± 0.15	5.7 ± 3.6 Å
5	BOPAM-SP5	−1.71	0.51 ± 0.15	5.7 ± 3.6 Å
6	BOPAM-SP6	−1.75	0.50 ± 0.15	5.8 ± 3.6 Å
7	BOPAM-SP7	−0.08	0.70 ± 0.12	2.6 ± 1.9 Å

*BOPAM-AB* Anti-*Acinetobacter baumanni* AMPs, *BOPAM-SP* Anti-*Streptocococcus pneumoniae* AMPs, *BOPAM-KP* Anti-*Klebsiella pneumoniae* AMPs

### Docking interaction analysis of the putative anti-pneumonia AMPs with bacterial pneumonia receptors

**Fig. 2:**
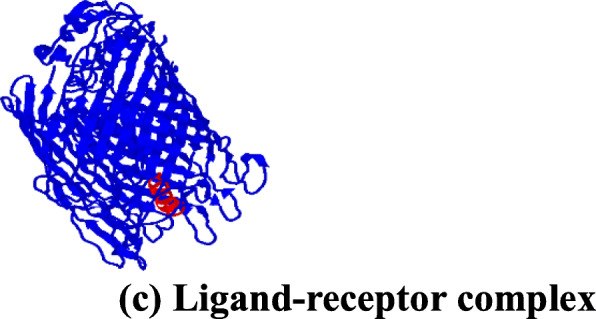
Representative of the docking interaction of a protein receptor and putative anti-pneumonia AMPs produced by PATCHDOCK and visualized using PyMol. BOPAM-AB3, 2, and 4 had higher binding affinity than all anti-Acinetobacter baumannii AMPs, BOPAM-SP2, and 6 had higher binding affinity than all anti-*Streptococcus pneumoniae* AMPs whilst BOPAM-KP2 and 6 had higher binding affinity than all anti-*Klebsiella pneumoniae* AMPs

The output images from the PATCHDOCK server after predicting the docking interaction between the anti-pneumonia AMPs (ligands) and the protein receptors were analysed (Fig. 2). The spatial docking interaction analysis showed that all the AMPs bound tightly to their respective proteins, indicating significant diagnostics since the geometry binding scores were higher than 8741 [25].

## Discussion

Only experimentally validated AMPs were retrieved from the various databases since they have proven activity against the target pathogens with minimum inhibitory concentration (MIC) as indicator using the agar dilution or broth microdilution methods [26]. Experimentally validated AMPs were used since their activities have been proven. These activities would be retained in the newly retrieved AMPs since HMMER creates a profile by retaining the functionally significant amino acid residues. The list of anti-pneumonia AMPs was retained within their respective pathogenic target groups as retrieved from the various databases to allow for specific species/pathogen profile creation. This step prevents the profile being sensitive to small misalignments and to report reliable E-values in order to capture the diversity of the sequences since the AMPs were derived from different organisms [27]. Clusters by HMMER also allow a minimum amount of similarity between all peptides. A total of three AMP profiles were created for each of the following classes (anti-*Streptococcus pneumoniae* (SP)*,* anti-*Acinetobacter baumannii* (AB) *and* anti-*Klebsiella pneumoniae* (KP)).

The profiles were tested using positive and negative datasets where the strength of the created profiles lies in its high discriminatory power. It is assumed that the AMPs used for the construction of the respective profiles have known anti-pneumonia activity as seen by the experimental evidence provided by the literature [26]. This independent testing was done with the negative dataset (neuro-peptides) to confirm whether the trained profiles would discriminate against non-anti-pneumonia peptides. This method of taking random sequences as a negative dataset is a routinely used procedure [28] and this is based on the assumption that probability of finding random sequences with a highly discriminative profile is very low.

In Table 2, AB had all its testing datasets as true positive but KP had 10 of its 35 testing datasets as true positive while SP had one of its four testing as true positive. All the anti-bacterial profiles discriminated against the neuropeptides as it was expected. The lower hit observed for the KP and SP profiles is as a result of an overlap of homologous relationship in the AMPs used in their profiles [29]. HMMER used a default E value of 0.05 for every hit considered to be true positive. All anti-bacterial profiles yielded true positive with E values lower than 0.05 indicating that there was only a 5% chance that the hit was false or random, i.e. true positive with an E value less than 0.05 is considered ideal, indicating an extremely high confidence.

Specificity estimates true negative rate by calculating the proportion of the negative datasets that were correctly predicted [30]. A poor specificity results when AMP profiles have closely related functions with other profiles. The specificity results for the profiles indicate that they did not have closely related functions with any other profiles. Sensitivity is the measure of the true positive rate which represents the ratio of the correctly predicted positive datasets to the total number of the positive datasets [31]. Low or moderate sensitivity is only possible where a false-positive might score higher than a true-positive. In this case, however, the model discriminated against all false-positive but some true-positive in KP and SP behaved as true-negative, strengthening further the case of overlapping consensus in the conserved domains. Accuracy considers interactions between features of AMPs by discriminating them against non-AMPs, even from similar accuracy values in other machine learning methods [32]. The accuracy results were very high for all the profiles indicating elimination of errors by nullifying misclassified AMPs from both positive and negative datasets. MCC is considered as the most robust parameter for evaluating the prediction of profiles. This is because it offers an advantage by increasing the understanding of the relationship between sensitivity, specificity and accuracy; reduces uncertainty through identification of profiles that are defective significantly; and searches for errors in the model [33]. This result agrees with the work of Bhadra, Yan [34] where performance was compared in terms of accuracy, precision, Mathew and Correlation Coefficient (MCC) using benchmark datasets as inputs.

A final list of AMPs was identified and the AMPs were ranked according to their E-values with those having the smallest E-values considered the most likely putative anti-pneumonia AMPs. There was a very low probability that these peptides were wrongly predicted to be anti-pneumonia AMPs. The consequence of the presence of charged, polar, and non-polar amino acids to the anti-bacterial putative AMPs is the conferment of charge, reduced or increased hydrophobicity, and reduced or increased binding potential on them. The resultant effect of reduced hydrophobicity on the non-polar face of the amphipathic helix of BOPAM-KP4, BOPAM-SP1, and 2 is poor peptide helicity, reduced self-associating ability in aqueous environments, and poor antimicrobial activity. Peptides with higher hydrophobicity would penetrate deeper into the hydrophobic core of the bacterial membrane, causing stronger hemolysis by forming pores or channels [35]. Thus, the antibacterial AMPs with increased hydrophobicity could potentially penetrate the membrane core. Reduced hydrophobicity is a consequence of polar amino acids. Recently, AMPs from sugar-functionalized phosphonium polymers have been reported to require the hydrophilic domains in their molecular structure to exert anti-bacterial activities against Gram-negative *Escherichia coli* and Gram-positive *Staphylococcus aureus* [36]. Cationic AMPs are said to be positively correlated with increased anti-microbial activities (Table 5). Therefore all the anti-bacterial AMPs which were positively charged indicated conformity with ideal AMPs with improved anti-microbial activities [37]. Isoelectric potential (pI) of peptides is a function of individual amino acids in both backbone groups. At a pH below the pI, AMPs carry a net positive charge and vice versa. A more hydrophilic peptide tends to have a more positive index. However, the tendency of some peptides to have a positive Boman index has been reported with the ability to detect HIV in a lateral flow device [12]. The use of physicochemical parameters as indices to evaluate AMPs is in agreement with the work of Hollmann, Martinez [38] where a re-evaluation of the physicochemical properties of antimicrobial peptides was investigated, resulting in a characteristic thermal transition profile in model vesicles which was used to categorize novel molecules with unknown biological activity.

*Acinetobacter baumannii* iron-regulated outer membrane protein has a strong potential as a receptor for the diagnosis of pneumonia caused by this organism. The protein is well-conserved throughout evolution and stable in vitro as indicated by its instability index of 25.61 (see Table 6). A protein whose instability index is smaller than 40 is predicted as stable in vitro. Studies have shown that the outer membrane protein (Omp) of *Acinetobacter baumannii* can be used as candidate bio-molecules in animal models for detection tests [39]. This protein possesses attributes such as outer membrane localization, high adhesion probability (0.53), possession of a single transmembrane helix and absence of homology to the human protein, presence of B-cell and T-cell epitopes binding with the high affinity of 40% survival rate for passive immunization and 20% for active in its outer membrane protein and have been explored for in silico technology to select promising diagnostic candidate [40]. The most significant recent developments in the diagnosis of pneumonia have occurred with antigen detection assays using pneumolysin [41]. The use of pneumolysin is very essential for *S. pneumoniae* diagnosis because it is produced in high concentration and stable in different body fluid samples across virtually all clinical isolates. Besides, based on the protein’s physicochemical properties using the charge, instability index, and half-life, pneumolysin is an attractive candidate receptor for the diagnosis of *Streptococcus pneumoniae. The* iron-regulated outer membrane protein is being used for the detection of *K. pneumoniae* using antibody detection because it is well-conserved throughout evolution and stable across clinical samples. *The use of receptor protein candidates such as Klebsiella pneumoniae iron-regulated outer membrane protein* [42]*, Acinetobacter baumannii iron-regulated outer membrane protein* [43]*, Streptococcus pneumoniae pneumolysin* [44]*, in the diagnosis of pneumonia is justified because they are produced in relatively high amount inside body fluid across all strains and subtypes of these pathogens; do not change with time; highly accessible either as cell surface receptor and relatively stable in a mild* in vitro *handling. These receptors were therefore retained for further analysis.*

Following the prediction of the putative anti-pneumonia peptides, it still had to be concluded whether these sequences can be considered *bona vide* AMPs. To classify these sequences as *bona vide* AMPs it had to conform to known AMPs in terms of characteristics as well as structure. As seen in the physicochemical characterization section of the sequences which was carried out on the predicted peptides indicating that the peptides conform to known AMPs. In this section, the structures of the peptides were predicted and the results indicates that these peptides conform to known AMPs using structure as the measurement. Taken together, it can be concluded that based on their characteristics and structure, these peptides can be considered *bona vide* AMPs. However, the AMPs are still considered putative anti-pneumonia peptides due to lack of experimental evidence for these molecules currently.

The C score value of the putative anti-pneumonia peptides 3-D structures indicate they have already been solved indicating existing templates within protein databases for use by I TASSER for their structural prediction except for BOPAM-KP2, 3, 4, BOPAM-SP3, 4. TM-score, on the other hand, is a recently proposed scale for measuring the structural similarity between two structures [45]. AMPs with TM-scores higher than 0.5, signifies structural similarity with the templates that were used to predict their structures [23, 24]. All anti-bacteria AMPs having RMSD within the accepted range (see Tables 7, 8, 9, and 10) had less distance and the atomic deviation between the superimposed peptides and the templates, which were used for their 3-D structure prediction [46, 47]. The purpose of proposing TM-score is to solve the problem with RMSD, which is sensitive to local error since RMSD is an average distance of all residue pairs in two structures. For instance, a misorientation of the structure will give rise to a big RMSD value although the global topology of the structure is correct. TM-score is not sensitive to misorientation in the distance of the residues which makes the score insensitive to the local modeling error and thus a more reliable measure. Both TM and RMSD scores are known standards for measuring structural similarity between two structures for accuracy of structure modeling when the native structure is known [24]. The C-score is a metric developed for I-TASSER to estimate the confidence of the modeling, however, when the native structure is unknown, it becomes imperative to predict the quality of the modeling, that is, the distance between the predicted model and the native structures. This is the reason for calculating the TM and RMSD scores of the predicted models relative to the native structures which are based on the C-scores.

Using this criteria of binding geometric score from Table 11 above, BOPAM-AB3, 2 and 4 would be better diagnostic molecules for the detection of *Acinetobacter baumannii* since these peptides showed the highest binding geometry scores compared to any other putative anti-*Acinetobacter baumannii* AMP. In addition, BOPAM-SP2 and 6 (Table 11) would be the better diagnostic molecules against *Streptococcus pneumoniae* based on the peptides observed binding geometry scores to this organism’s identified receptor. Furthermore, BOPAM-KP2 and 6 (Table 11) showed the highest binding geometry scores to the *Klebsiella pneumoniae* identified receptor than any other BOPAM-KPs.

**Table 11:** Quality assessment scores of the docking analysis for the anti-pneumonia putative AMPs and the pneumonia receptors

S/N	Receptors	Ligands	Binding Scores
1	Iron regulated outer membrane protein	BOPAM-KP2	13,036
2	Iron regulated outer membrane protein	BOPAM-KP3	12,384
3	Iron regulated outer membrane protein	BOPAM-KP4	12,960
4	Iron regulated outer membrane protein	BOPAM-KP5	10,810
5	Iron regulated outer membrane protein	BOPAM-KP6	13,208
6	Iron regulated outer membrane protein	BOPAM-KP7	12,092
7	Pneumolysin	BOPAM-SP1	12,306
8	Pneumolysin	BOPAM-SP2	13,606
9	Pneumolysin	BOPAM-SP3	12,116
10	Pneumolysin	BOPAM-SP4	12,384
11	Pneumolysin	BOPAM-SP5	12,514
12	Pneumolysin	BOPAM-SP6	13,306
13	Pneumolysin	BOPAM-SP7	11,830
14	Iron Regulated OMP	BOPAM-AB1	10,566
15	Iron Regulated OMP	BOPAM-AB2	11,806
16	Iron Regulated OMP	BOPAM-AB3	12,480
17	Iron Regulated OMP	BOPAM-AB4	11,802

## Conclusions

AMPs have shown great promise in circumventing the drawbacks associated with the current diagnostic systems. Eighteen putative AMPs were identified from the use of HMMER for these bacterial pathogens for use in the differential diagnosis. This research offers new insights into the in silico modular architecture, the evolution of host defense molecules containing core motifs, and diagnostic helices associated with antimicrobial activity of putative AMPs functions against bacterial pneumonia pathogen. The main goal of this in silico diagnostic system is to ease the search for early detection. This would assist medical practitioners towards the correct treatment plan and subsequently, enable patients to develop accommodating lifestyles. This research work could be pursued for molecular validation through the binding of these AMPs with the bacterial proteins respectively, using an “on/off” binding experiment in an LFD setting to develop a prototype with these specific AMPs conjugated to gold nanoparticles (AuNPs) to accurately and sensitively detect the viral and bacterial pathogens within patient samples. Future work will include the site-directed mutagenesis of the putative AMPs to optimize them into more potent candidate diagnostic molecules. This would be followed by an in vitro study of the anti-pneumonia activity of the mutated peptides. Also, the EC_50_ of all the AMPs and their therapeutic or selective index will be assessed for the optimized AMPs. The anti-pneumonia activity of these AMPs will be carried out on different pseudotypes of the pneumonia pathogens to determine their broad-spectrum activity. Finally, the binding complex formed between the pathogen receptors and putative AMPs will be solved using structural biology to validate the observations made by the in silico binding study.

## Methods

### Data retrieval (literature mining)

The experimentally validated anti-pneumonia AMPs for the bacterial causative agents (*Streptococcus pneumoniae, Acinetobacter baumannii, and Klebsiella pneumoniae*) were retrieved manually from antimicrobial peptide databases such as Antimicrobial Peptides Database (APD) [48, 49], Collection of Antimicrobial Peptides (CAMP) [50]. Thereafter, curation was carried out through literature mining to confirm that all the retrieved AMPs were either experimentally validated or predicted. Duplicate experimentally validated AMPs were then discarded from the list using the Cluster Database at High Identity with Tolerance (CD-HIT) [51].

### Training and testing datasets (data mining)

The final list of the experimentally validated AMPs was categorized according to their specific pathogenic agents with AB denoting anti-*Acinetobacter baumannii*; SP - anti-*Streptococcus pneumoniae*; KP - anti-*Klebsiella pneumoniae*. Each category of the aforementioned datasets was randomly divided into two portions: three-quarters of each data set was utilized as the training set (to build each profile), whilst one-quarter was used as the testing dataset (for optimization/calibration of the created profiles).

### Construction of AMPs profiles (text mining)

The Hidden Markov Models (HMMER) algorithm version 2.3.2 [52] was used to construct specific pathogen-targeted models/profiles using the respective training datasets. All the HMMER profiles were built on the Ubuntu 12.04 LTS operating system. The task was accomplished on a terminal and the command lines used to build each profile was written following the corresponding algorithm and the steps involved in their construction were as below:

For the first step, the training datasets of each target class were aligned using the ClustalW alignment tool [53]. The alignment was carried out using the command line:

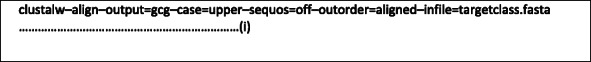



The command line simply states <<do an alignment of the sequences which are in the upper case found in the input file “target class.fasta” with the FastA, using ClustalW as multiple alignment tools and GCG Postscript output for graphical printing>>. The output of the command results in the construction of aligned sequences, called “target class.msf”. The aligned sequences were used as input in the next step.

The next step enhances the construction of the profiles of the target class sequences by showing the common motifs/signatures within the profiles. To achieve this, the “Build profiles” was run using the following command:

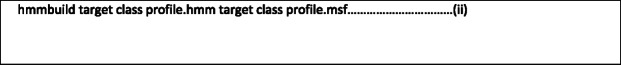



To enhance the sensitivity of the profiles, the file generated (target class. hmm) from the profile building step was calibrated by using the command line:

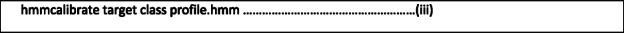



The resulting profiles “target class.hmm” was used in evaluating the performance of the profiles by testing the created profiles on an independent AMP dataset.

### Independent profile testing

The independent testing of each created profile was performed in a step called “Query profiles”. The testing and negative datasets were queried against the created profiles using the command line, with an E-value threshold of 0.05:

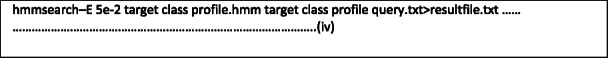



### Performance measurement of each profile

Statistical performance measures were then calculated using sensitivity, specificity, accuracy and Mathew Correlation Coefficient as parameters. The measures used were employed accordingly.

### Scanning the profiles for motifs discovery across the proteome sequences

Proteome sequences were scanned by the respective profiles/predictive profiles with the list of all proteome sequences (in the fasta format) retrieved, from the Ensembl database (http:// www.ensembl.org/index.html) and the UniProt database (http://www.uniprot.org/) for the identification of putative anti-pneumonia AMPs. A cut-off E-value was set to be 0.05 for the search of putative anti-pneumonia AMPs. This was accomplished using “hmmsearch” module of the HMMER algorithm with the command line employed stated below:

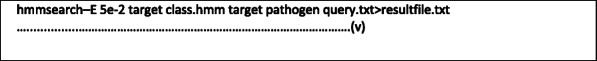



Where specific target class.hmm in one of the six profiles, target class query.txt representing the species scanned against the profile, and resultfile.txt is the result file saved after querying that species against a particular pathogen profile.

### Identification of receptors

Bacterial receptors, such as cell surface receptors, were identified for the bacterial causative agents (*Streptococcus pneumoniae, Acinetobacter baumannii, and Klebsiella pneumoniae*) implicated in pneumonia to serve as targets for the identified AMPs.

#### Protein retrieval

Bacterial pneumonia proteins were collected from various protein data banks (PDB) such as National Centre for Biotechnology Information (NCBI), UniProt, Google Scholar, and Ensembl through literature mining. Thereafter, curation was performed to verify that all the retrieved pathogen pneumonia proteins were complete or partial. Partial proteins were discarded and complete protein was kept for further analysis. BLAST analysis was performed using the UniProt interface for further assurance of specificity with E threshold of 0.01 such that the bacterial pneumonia proteins of interest were not present in other bacteria and viruses.

### Physicochemical properties of the putative anti-pneumonia AMPs and the pneumonia proteins

Physicochemical properties of the putative anti-pneumonia AMPs and their respective protein receptors were calculated using the prediction interface of Bactibase (http://bactibase.pfba-lab-tun.org/physicochem) [54, 55] and APD ((http://aps.unmc.edu/AP/design/design_improve.php)) [48, 49] using the amino acid sequences of the putative peptides and receptor proteins as input.

### De novo structure predictions of the putative anti-pneumonia AMPs and pneumonia proteins (ligands)

Prediction of the top ranking putative anti-pneumonia AMPs structures, based on their predictive E-values, as well as the structure of pneumonia bacterial proteins was performed using I-TASSER (Iterative Threading ASSembly Refinement) server, which is an example of a de novo method of peptide or protein structure prediction (Eswar and Sali, [56]). In brief, the 3-D structures of the anti-Pneumonia AMPs and specific bacterial pneumonia protein was predicted by uploading each sequence onto the I-TASSER website. The users enters their email addresses into which the results link will be sent. After, naming the uploaded sequence, the menu “Run I-TASSER” was selected (Roy et al., [24]). The 3-D structures of the AMPs and their respective protein receptors were visualized using the PyMOL version 1.3. This was achieved by downloading the latest version of the PyMol on Ubuntu Linux, extracting and installing it using the terminal command line.

### Docking analysis of the putative anti-pneumonial AMPs and Pneumonial proteins

The docking of the anti-bacterial AMPs to their respective pneumonia proteins was carried out using PatchDock Beta 1.3 version, a free online web-server that enables the docking of the protein-small ligand molecule, available at http://bioinfo3d.cs.tau.ac.il/PatchDock/ (Schneidman-Duhovny et al., [57]). In brief, the 3-D structures of the anti-bacterial pneumonia putative AMPs and the bacterial pneumonia protein receptors PDB files from I-TASSER onto the PatchDock server website, after which the user enters an email address. The cluster RMSD was set to 4.0 Å and the “protein-small ligand” was selected for complex type. The task was submitted by selecting “the Submit Form”. The docking results were deposited via an email notification containing the web link to the docking results. Interaction analysis of the complex formation between the anti-bacterial pneumonia putative AMPs and their respective pneumonia protein receptors was done using PyMol version 1.3.

## Data Availability

All relevant data are enclosed in the manuscript.

## Abbreviations

POC: Point-of-care
HMMER: Hidden Markov Models
AMPs: Antimicrobial peptides
CRP: C-reactive protein
PCT: Procalcitonin
STREM-1: Soluble triggering receptor expressed on myeloid cells-1
CD163: Cluster of differentiation 163
HMGB-1: High mobility group box-1
HIV: Human immuno-deficiency virus
CD-HIT: Cluster Database of High Identity with Tolerance
CAMP: Collection of antimicrobial peptide database
APD: Antimicrobial peptide database
BACTIBASE: Bacteria database
UNIPROT: Universal protein database
TP: True positive
TN: True negative
FP: False-positive
FN: False-negative
AB: Anti-*Acinetobacter baumannii* profile
SP: Anti-*Streptococcus pneumoniae* profile
KP: Anti-*Klebsiella pneumoniae* profile
AuNP: Gold nanoparticle
I-TASSER: Iterative Threading Assembly Refinement
RMSD: Root mean square deviation
TM score: Template Modelling score
C-score: Confidence score
LFD: Lateral flow device
